# Water channel changes in astrocytes associated with buprenorphine administration in a rat model of diffuse traumatic brain injury

**DOI:** 10.21203/rs.3.rs-9152535/v1

**Published:** 2026-03-30

**Authors:** Radina L. Lilova, Tytus Bernas, Jane Ryu, Corrina Kelliher, Audrey Lafrenaye

**Affiliations:** Virginia Commonwealth University School of Medicine; Virginia Commonwealth University School of Medicine; Virginia Commonwealth University School of Medicine; Virginia Commonwealth University School of Medicine; Virginia Commonwealth University School of Medicine

**Keywords:** Diffuse traumatic brain injury, buprenorphine, opioid, GFAP, SWELL1/LRRC8A, TRPM4, AQP4, astrocyte changes, cFPI, rat

## Abstract

Traumatic brain injury (TBI) is a significant concern, with millions sustaining a TBI each year. The vast majority are categorized as mild, in which diffuse pathologies are the hallmark and multiple cell types are affected, including astrocytes. Buprenorphine (bup), a semi-synthetic opioid, is a common human and veterinary analgesic. Previous studies from our group showed that a single dose of slow-release bup administered acutely following TBI alters astrocyte morphology at 1 day and 4 weeks post-injury in a region-specific manner. The current study expands on these findings by examining how four major astrocyte-associated proteins, GFAP, SWELL1/LRRC8A, TRPM4, and AQP4, are affected by such a dose of bup. Adult male rats received either sham or TBI via a central fluid percussion injury model, followed by saline or bup 15 min following injury. Protein quantification at 1 day and 4 weeks post-injury revealed region- and time-dependent effects of acute bup administration. Specifically, bup reduced hippocampal GFAP, increased cortical TRPM4, and elevated thalamic AQP4 and SWELL1 at 4 weeks following TBI. Taken together, these findings indicate that a single post-injury dose of bup can alter major astrocytic protein expression beyond the acute phase in a region- and time-dependent manner.

## Introduction

Traumatic brain injury (TBI) is a significant public health concern with no definitive cure. Globally, between 27 and 69 million individuals will sustain a TBI each year, with roughly 80% of those cases falling into the category of mild TBI^1–3^. Even with what are labelled “mild” injuries, TBI survivors can endure an array of cognitive^4–6^, emotional^7–9^, sensory^10^, and physical^11–13^ consequences that impair their quality and length of life both acutely and chronically^14–16^. In the United States, the economic consequences from nonfatal TBI alone were estimated at nearly $41 billion in 2016^17^, further underscoring the burden TBI puts on both patients and the broader healthcare system.

TBI pathologies can be subdivided into focal lesions, in which the area of tissue damage is evident, and diffuse pathologies, in which there is no specific injury locus, but rather injured cells are dispersed throughout the parenchyma in clusters throughout various brain regions. While focal lesions and diffuse pathologies are distinct, these two pathologies can often coexist following a TBI. Diffuse pathologies are highly associated with what are clinically categorized as mild TBIs, and because the vast majority of TBIs are indeed mild, these pathologies are of great interest to ongoing research. More difficult to pinpoint, the hallmark of such diffuse pathologies is oftentimes progressive diffuse axonal injury (DAI) along with prolonged glial activation, which can persist for weeks, months, and sometimes even years following a TBI. Astrocytic responses, in particular, have been recognized as highly heterogeneous and temporally dynamic, with both protective and maladaptive roles in post-TBI tissue remodeling^18–21^.

Astrocytes are a heterogeneous population of glial cells involved in numerous processes essential for proper brain function, including edema resolution, glymphatic flow, and maintenance of the blood-brain barrier^22^. Among the many proteins expressed by astrocytes, several are particularly well-established as key regulators of astrocytic structure, volume, and ion-water homeostasis following injury. Glial fibrillary acidic protein (GFAP) is the most widely studied astrocytic marker. As a type III intermediate filament enriched within main processes and perivascular endfeet of astrocytes. Upregulation of GFAP reflects cytoskeletal remodeling abilities of reactive astrocytes and may contribute to altered perivascular architecture and disrupted brain fluid dynamics^23^. SWELL1/LRRC8A is a core subunit of the volume-regulated anion channel and has been shown to be highly involved in regulatory volume decreases in various cell types, including astrocytes^24^. Transient receptor potential melastatin 4 (TRPM4) is a Ca^2+^-activated, nonselective cation channel that is expressed in astrocytes and endothelial cells and functions to enhance water influx into the cell^25,26^. Aquaporin-4 (AQP4), the predominant astrocytic water channel, is normally enriched in perivascular endfeet where it supports bidirectional water transport between interstitial and perivascular spaces^27^. Fluid movement regulated by interplays among these channels are key for proper solute clearance in the brain by the glymphatic system^28–30^.

Buprenorphine (bup) is a common analgesic used in human and veterinary medicine as well as in pre-clinical animal research. Pharmacologically, bup is a semi-synthetic opioid derived from thebaine. It functions as a high-affinity partial agonist at the μ-opioid receptor (MOR), an antagonist at the κ-opioid receptor (KOR), and also has some weak partial agonist activity at nociceptive receptors (ORL-1).^31^ This receptor activation profile can provide effective pain management while limiting some of the adverse effects associated with full μ-opioid agonists, such as respiratory depression and profound euphoria, and has contributed to its adoption for both perioperative and chronic pain management^32,33^. Beyond its analgesic properties, bup has been shown to modulate neuroinflammatory signaling, astrocytic activation, and blood-brain barrier integrity through its actions on MORs and related immune pathways^34–37^.

Previous studies from our group found that a single dose of slow release bup given acutely after a TBI changes the morphology of astrocytic cells 1 day and 4 weeks following injury in a region-specific manner^38,39^. These findings prompted the current investigation into bup’s potential effects on astrocyte-associated protein expression and glymphatic function both acutely and more chronically post TBI.

## Results

### Buprenorphine administration decreases TBI-induced GFAP expression in a region-specific manner

GFAP is a type III intermediate filament crucial for cytoskeletal support of astrocytes, among other roles. GFAP is well established to be upregulated following TBI and serves as a hallmark astrocytic marker^43^. Our group has previously reported region-specific morphological changes in GFAP-positive astrocytes at both 1-day and 4-weeks post-TBI and bup treatment^38,39^. Therefore, in the current study we used western blotting to assess regional GFAP expression changes in the context of bup treatment at two time points post-cFPI in rats.

**Figure 1:**
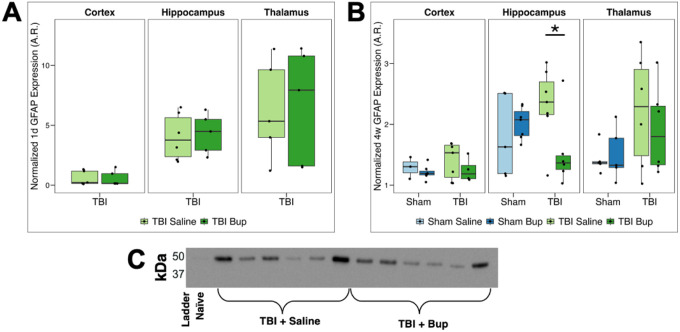
GFAP protein level changes associated with injury, timepoint, and buprenorphine treatment. (**A)** Normalized GFAP levels 1 day following cFPI-induced diffuse TBI in the right cortex, hippocampus, and thalamus. Saline-treated animals are shown in light green, and buprenorphine (bup)-treated animals are in dark green. **(B)** Normalized GFAP levels 4 weeks following either sham injury or cFPI-induced diffuse TBI in the right cortex, hippocampus, and thalamus. Sham/saline-treated animals are light blue, sham/bup-treated animals are dark blue, TBI/saline-treated animals are light green, and TBI/bup-treated animals are dark green. Median values and interquartile ranges are displayed for each cohort. Each dot represents individual animals. Asterisks indicate region-specific, within-injury, treatment-driven differences in GFAP expression (*p* < 0.05). **(C)**Representative chemiluminescent western blot with quantified band at ~50 kDa. Full uncropped blot and the corresponding total protein normalization image in **Figure S1**.

In 1-day post-cFPI rats, a two-way ANOVA revealed a main effect of region (*F*(2, 25) = 11.27, *p* < 0.001) but no effect of treatment and no region × treatment interaction. Post hoc comparisons showed higher GFAP levels in the hippocampus and thalamus relative to the cortex in both the saline- and bup-treated TBI animals 1 day post TBI (all *p* < 0.05), with no relative difference in GFAP levels between the hippocampus and thalamus (Fig. 1A; Table 1; **Table S1**).

At 4 weeks, a three-way ANOVA demonstrated significant main effects of injury (*F*(1, 64) = 6.17, *p* = 0.016) and region (*F*(2, 64) = 12.76, *p* < 0.001), but no main effect of treatment (Fig. 1B; Table 2). A significant injury × treatment interaction was observed in saline treated animals (*F*(1, 64) = 7.25, *p* = 0.009; Table 2) in which GFAP levels were elevated after TBI compared with the sham injury group (overall *p* = 0.006; Fig. 1; **Table S2**). This interaction was not recapitulated in the bup-treated animal groups. While an overall main effect of region was observed, post hoc tests showed no detectable regional differences across the sham groups (Fig. 1B; **Table S3**). In saline treated TBI rats, hippocampal GFAP levels were significantly higher than levels in the cortex (*p* = 0.006), with a non-significant trend for GFAP levels to also be higher in the thalamus compared to the cortex (*p* = 0.088; Fig. 1; **Table S3**). Bup treatment following TBI trended toward attenuation of the TBI-associated increase in GFAP levels observed in saline treated animals (overall *p* = 0.055), which was particularly apparent in the hippocampus (*p* = 0.04; Fig. 1B; **Table S4**), indicating that bup could mediate GFAP elevation in a region-specific manner following diffuse brain injury.

### Buprenorphine administration altered TBI-induced SWELL1 expression in a region-specific manner

SWELL1/LRRC8A is an essential component of the volume-regulated anion channel (VRAC) complex, implicated in osmotic regulation and cell volume homeostasis^24^. To determine whether SWELL1 expression is modulated by injury or bup administration, we analyzed regional protein levels at 1-day or 4-weeks post-cFPI TBI.

**Figure 2:**
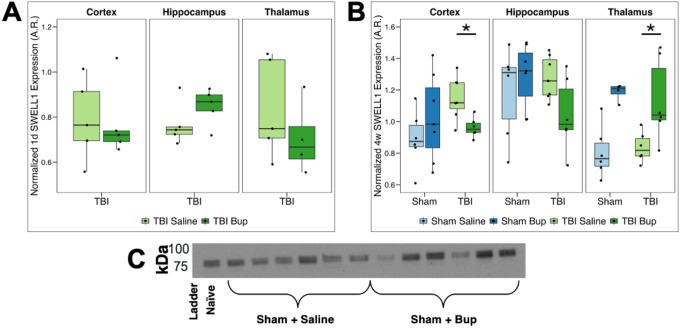
SWELL1 protein level changes associated with injury, timepoint, and buprenorphine administration. **(A)** Normalized SWELL1 **expression** 1 day following cFPI-induced diffuse TBI in the right cortex, hippocampus, and thalamus. Saline-treated animals are shown in light green and buprenorphine (bup)-treated animals in dark green. **(B)** Normalized SWELL1 expression 4 weeks following either sham injury or cFPI-induced diffuse TBI in the right cortex, hippocampus, and thalamus. Sham/saline-treated animals are light blue, sham/bup-treated animals are dark blue, TBI/saline-treated animals are light green, and TBI/bup-treated animals are dark green. Median values and interquartile ranges are displayed for each cohort. Each dot represents individual animals. Asterisks indicate region-specific, within-injury, treatment-driven differences in SWELL1 expression (*p* < 0.05). **(C)** Representative chemiluminescent western blot with quantified doublet band at ~85–105 kDa. Full uncropped blot and the corresponding total protein normalization image in **Figure S2**.

A two-way ANOVA showed no significant main effects of region or treatment at 1-day post-TBI (*p* > 0.1) (Fig. 2A; Table 3).

At 4 weeks following sham or TBI and saline or bup treatment, three-way ANOVA revealed a strong main effect of region (*F*(2, 64) = 10.68, *p* < 0.001) and a region × treatment interaction (*F*(2, 64) = 3.30, *p* = 0.043; Table 4). Post hoc testing indicated a trend toward elevated SWELL1 following TBI (overall *p* = 0.087; **Table S5**), particularly in the cortex (*p* = 0.017; Fig. 2B; **Table S5**). Overall, SWELL1 remained largely stable following injury, though regional expression patterns differed modestly across treatment conditions (Fig. 2B; Table 4; **Table S5-Table S7**). Regional comparisons demonstrated significantly lower SWELL1 in the thalamus compared to the hippocampus (*p* = 0.001) of sham animals treated with saline (Fig. 2B; **Table S6**). Following TBI and saline administration SWELL1 levels were significantly lower in the thalamus compared to either the cortex (*p* = 0.001) or the hippocampus (*p* < 0.001; Fig. 2B; **Table S6**). While a trend was observed toward higher SWELL1 in the hippocampus compared to either the cortex (*p* = 0.066) or the thalamus (*p* = 0.083) in bup treated sham controls, this regional trend was lost in TBI animals with bup treatment (**Table S6**). At 4 weeks following TBI and bup treatment there was a region-specific shift in SWELL1 expression levels in which SWELL1 was reduced in the cortex but increased in the thalamus following TBI and bup treatment (*p* = 0.037; Fig. 2B; **Table S7**).

### Buprenorphine administration decreases TBI-induced TRPM4 expression in a region-specific manner

TRPM4 is a calcium-activated, non-selective cation channel that contributes to membrane depolarization and cell swelling under pathological conditions^28^. Given its important role in astrocytic and vascular responses following brain injury, we assessed regional TRPM4 expression following bup treatment 1 day and 4 weeks post-cFPI TBI in rats. We visualized western blot bands at multiple molecular weights indicating both breakdown of TRPM4 (bands < 140 kDa) as well as glycosylated TRPM4 (band ~ 250 kDa), therefore, we quantified all bands of TRPM4 for our analysis.

**Figure 3:**
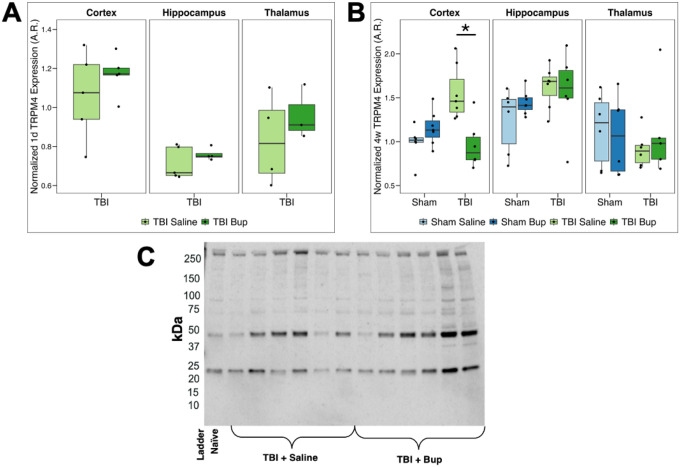
TRPM4 protein level changes associated with injury, timepoint, and buprenorphine administration. **(A)** Normalized TRPM4 expression 1 day following cFPI-induced diffuse TBI in the right cortex, hippocampus, and thalamus. Saline-treated animals are shown in light green and buprenorphine (bup)-treated animals in dark green. **(B)** Normalized TRPM4 expression 4 weeks following either sham injury or cFPI-induced diffuse TBI in the right cortex, hippocampus, and thalamus. Sham/saline-treated animals are light blue, sham/bup-treated animals are dark blue, TBI/saline-treated animals are light green, and TBI/bup-treated animals are dark green. Median values and interquartile ranges are displayed for each cohort. Each dot represents individual animals. Asterisks indicate region-specific, within-injury, treatment-driven differences in TRPM4 expression (*p* < 0.05). **(C)** Representative chemiluminescent western blot with quantified bands at ~30, 55, 85, and 200 kDa, summed together for overall expression level. Full uncropped blot and the corresponding total protein normalization image in **Figure S3**.

At 1-day post-injury, a two-way ANOVA revealed a main effect of region (*F*(2, 24) = 13.21, *p* < 0.001) but no effect of treatment (Fig. 3A; Table 5). Post hoc analyses showed higher TRPM4 protein levels in the cortex compared with hippocampus (*p* = 0.024) and a similar trend of higher TRPM4 in the cortex relative to thalamus (*p* = 0.089) in saline-treated TBI animals. This pattern of higher TRPM4 levels in the cortex compared to either the hippocampus (*p* < 0.001) or the thalamus (*p* = 0.027) was more apparent in bup-treated rats 1 day following TBI (Fig. 3A; Table 5; **Table S8**).

At 4 weeks following sham or TBI and saline or bup treatment, three-way ANOVA demonstrated a significant main effect of region (*F*(2, 64) = 5.00, *p* = 0.010), a trend toward an effect of injury (*p* = 0.057) and no effect of treatment (Fig. 3B; Table 6). However, a significant injury × region × treatment interaction was observed (*F*(2, 64) = 4.65, *p* = 0.013; Table 6). Post hoc testing revealed increased TRPM4 levels 4 weeks after TBI in saline-treated animals overall (*t*(35.82) = 2.18, *p* = 0.036), driven by higher cortical TRPM4 expression in TBI saline treated animals compared to sham controls (*p* = 0.002), while bup-treated animals showed no significant injury effect (Fig. 3B; **Table S9**). Regionally, there were no significant differences in TRPM4 levels in sham animals treated with saline, however, hippocampal TRPM4 at 4 weeks post sham and bup treatment was higher than cortical levels (*p* = 0.022; **Table S10**). At 4 weeks following TBI and saline treatment thalamic TRPM4 levels were significantly lower than cortical (*p* = 0.001) or hippocampal (*p* < 0.001) TRPM4 levels (Fig. 3B; **Table S10**). However, in TBI/bup animals cortical TRPM4 levels were significantly reduced compared to hippocampal TRPM4 expression (*p* = 0.022) and compared to TRPM4 levels in the cortex of saline treated animals 4 weeks following a cFPI TBI (*p* = 0.004; Fig. 3B; **Table S11**).

### Buprenorphine administration decreases TBI-induced AQP4 expression in a region-specific manner

Aquaporin-4 (AQP4) is a bidirectional water channel abundantly expressed in astrocytic endfeet, where it regulates water transport and contributes to edema formation following injury^22,28^. As with the other proteins of interest, to assess whether bup modulates AQP4 expression after diffuse TBI, western blotting was performed 1 day and 4 weeks post-cFPI in male rats.

**Figure 4:**
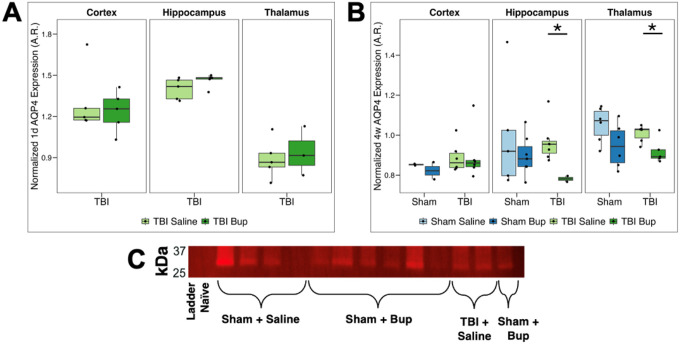
AQP4 protein level changes associated with injury, timepoint, and buprenorphine administration. **(A)** Normalized AQP4 expression 1 day following cFPI-induced diffuse TBI in the right cortex, hippocampus, and thalamus. Saline-treated animals are shown in light green and buprenorphine (bup)-treated animals in dark green. **(B)** Normalized AQP4 expression 4 weeks following either sham injury or cFPI-induced diffuse TBI in the right cortex, hippocampus, and thalamus. Sham/saline-treated animals are light blue, sham/bup-treated animals are dark blue, TBI/saline-treated animals are light green, and TBI/bup-treated animals are dark green. Median values and interquartile ranges are displayed for each cohort. Each dot represents individual animals. Asterisks indicate region-specific, within-injury, treatment-driven differences in AQP4 expression (*p* < 0.05). **(C)** Representative fluorescent western blot with quantified bands at ~28 kDa. Full uncropped blot and the corresponding total protein normalization image in **Figure S4**.

At 1 day post-injury, two-way ANOVA revealed a significant main effect of region (*F*(2, 24) = 34.90, *p* < 0.001) but not of treatment (Fig. 4A; Table 7). Post hoc analyses showed greater AQP4 expression in cortex (*p* = 0.014) and hippocampus (*p* < 0.001) compared with thalamus in both saline- and bup-treated TBI animals (Fig. 4A; Table 7; **Table S12**). While thalamic and cortical AQP4 levels remained relatively consistent 1 day following TBI and bup treatment, hippocampus AQP4 levels increased leading to significant regional differences between hippocampus and either cortex (*p* = 0.024) or thalamus (*p* = 0.002) at 1 day following TBI and bup treatment (Fig. 4A; **Table S12**).

At 4 weeks following sham or TBI and saline or bup treatment, a three-way ANOVA identified significant effects of region (*F*(2, 63) = 14.30, *p* < 0.001) and treatment (*F*(1, 63) = 8.71, *p* = 0.004), and an injury × region interaction (*F*(2, 63) = 6.56, *p* = 0.003) (Fig. 4B; Table 8). Within saline-treated animals, AQP4 levels were only elevated in the cortex following TBI (*p* = 0.024; **Table S13**). Bup-treated rats exhibited divergent injury effects in different brain regions, with higher cortical AQP4 expression at 4 weeks following TBI compared to sham (*p* = 0.023) but reduced hippocampal AQP4 levels after TBI compared to their bup-treated sham counterparts (*p* = 0.002; Fig. 4B; **Table S13**). Regionally, thalamic AQP4 was higher than cortical levels across most groups (Fig. 4B; Table 8; **Table S14**). The only group in which AQP4 levels were not higher in the thalamus compared to the cortex was the TBI/bup group (**Table S14**). Hippocampal levels of AQP4 were not significantly different from thalamic or cortical levels in saline treated animals regardless of injury, consistent with a gradient of expression independent of injury (Fig. 4; **Table S14**). Bup treatment did not impact AQP4 levels in sham animals in any brain region, however, there was a trend toward lower AQP4 expression in the thalamus (*p* = 0.095; **Table S15**). Treatment with bup did not alter AQP4 levels in the cortex 4 weeks following TBI, however bup treatment significantly reduced AQP4 levels in both the hippocampus (*p* < 0.001) and thalamus (*p* = 0.031) compared to saline-treated TBI animals (Fig. 4; **Table S15**), indicating a bup-mediated reduction in AQP4 following TBI.

### Buprenorphine treatment trends toward reductions in long-term solute clearance post-TBI

**Figure 5:**
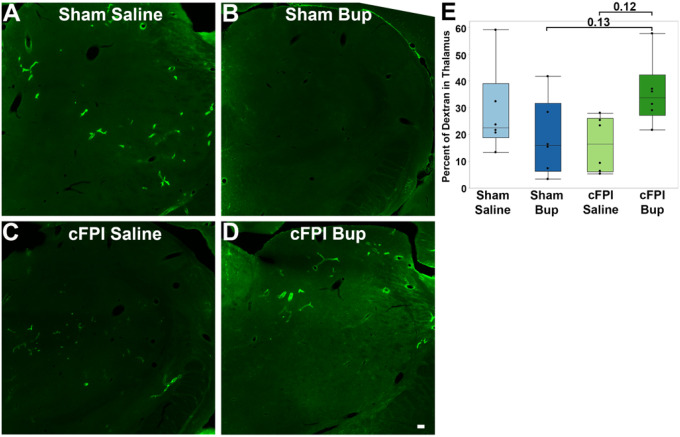
Parenchymal retention of fluorescently tagged dextran in the thalamus at 4 weeks following TBI and bup treatment. **(A-D)** Representative micrographs of intracerebroventricularly infused 10-kDa fluorescent dextran (green) within the thalamus from **(A)** sham animals treated with saline, **(B)** sham animals treated with buprenorphine (bup) administered 15 min post-procedure, **(C)** animals subjected to central fluid percussion injury (cFPI) followed by saline treatment, and **(D)** animals subjected to cFPI followed by bup treatment. **(E)** At 4 weeks post-injury the percent of tagged dextran contained within the thalamus was increased following cFPI with bup (dark green bars) compared to sham-bup (dark blue bars) or cFPI animals following saline administration (light green bars). Each point represents a separate animal.

Alterations in AQP4 expression have been linked to impaired parenchymal solute clearance. Based on our finding of reduced AQP4 expression in the thalamus 4 weeks after cFPI in bup-treated animals, we directly assessed solute clearance within the thalamus. To do this, we infused fluorescently tagged 10-kDa dextran into the lateral ventricle and quantified the percentage of the thalamic region of interest containing fluorescent signal 2 hours following infusion. A two-way ANOVA (injury × drug) revealed a significant interaction between injury and treatment in the thalamus (*F*(1,20) = 6.94, *p* = 0.016). Post hoc comparisons demonstrated increased parenchymal dextran burden in bup-treated TBI rats relative to both saline-treated injured rats (Student’s t-test *p* = 0.015; BH-adjusted *p* = 0.13) and a strong trend with sham rats receiving bup (Student’s t-test *p* = 0.053; BH-adjusted *p* = 0.13; Fig. 5). However, neither of these pairwise comparisons remained significant following correction for multiple testing, the interaction effect suggests that bup treatment may exacerbate impairments in parenchymal solute clearance following TBI.

### AQP4-GFAP colocalization following injury and buprenorphine treatment

To determine whether changes in AQP4 expression were accompanied by altered localization, spatial associations between AQP4 and GFAP were quantified in the thalamus 4 weeks after cFPI in animals treated with saline or bup. Intensity-based colocalization metrics were calculated using GFAP-positive and AQP4-positive pixels above background within 3D reconstructions of two hemi-thalami per animal. Quadrant-based frequency analyses of 3 × 3-pixel neighborhoods revealed substantial variability in AQP4-GFAP spatial relationships both within and between animals, reflecting heterogeneous patterns of protein distribution across thalamic subregions.

**Figure 6:**
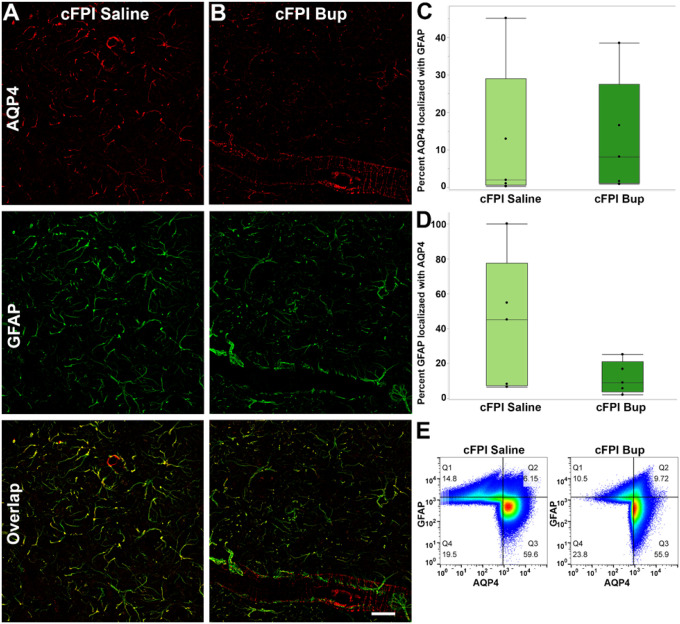
Localization of AQP4 is consistent following injury regardless of bup treatment. **(A&B)** Representative micrographs of thalami from **(A)** animals sustaining a central fluid percussion brain injury (cFPI) followed by saline administration or **(B)**animals sustaining a cFPI followed by bup administration immunolabeled with AQP4 (red) and GFAP (green). **(C)** The percentage of total AQP4 pixels that colocalized with GFAP and **(D)** the percentage of total GFAP-positive pixels that colocalized with AQP4 was consistent in animals treated with saline (light green bars) and cFPI animals treated with bup (dark green bars). Each dot represents a separate animal. **(E)** 4 weeks post-cFPI colocalization scatter plots of all GFAP-positive and AQP4-positive pixels collated from all saline (left plot) or bup (right plot) treated TBI animals, indicated a greater number of high intensity pixels in the AQP4 quadrant (Q3) in saline treated cFPI thalamus compared to bup treated cFPI animals. Scale bar = 20 mm.

Colocalization of AQP4 with GFAP was evaluated to determine whether buprenorphine treatment altered AQP4 localization within astrocytic processes following TBI. The fraction of AQP4 signal overlapping with GFAP did not differ between groups (saline 0.12 ± 0.18 vs. bup 0.13 ± 0.15; Student’s t-test *p* = 0.94; Fig. 6C). However, despite variability across animals, there was a trend toward reduced colocalization of AQP4 within GFAP-positive astrocytic processes in bup-treated animals compared with saline-treated animals (mean ± SD: saline 0.43 ± 0.43 vs. bup 0.12 ± 0.09; Student’s t-test *p* = 0.12; Fig. 6D). Additionally, consistent with our western blot findings, AQP4-positive pixels were less intense overall in bup-treated animals compared to saline controls (Fig. 6E). These findings suggest that although total AQP4 levels are reduced following TBI and bup treatment, the remaining AQP4 may be preferentially redistributed away from astrocytic processes and potentially toward perivascular domains.

## Discussion

Traumatic brain injury (TBI) triggers a complex cascade of cellular and molecular events, and damage extends well past the initial mechanical insult to the brain. One of the chief cell types involved in TBI response are astrocytes, which can activate, remodel their cytoskeleton, alter expression of water and ion channels, and regulate extracellular fluid dynamics following an insult. As astrocytes serve a multitude of functions to promote brain homeostasis, it is not surprising that these are highly heterogeneous cells with variable morphological and expression profiles^44–47^. Astrocytes have been subdivided based on their morphological presentation into grey matter populating protoplasmic astrocytes, that have long branching processing and complex process networks, and white matter populating fibrous astrocytes with short, highly branching process networks for decades^44^. Heterogeneity of astrocytes varies regionally across the CNS, with distinct molecular, morphological, and functional profiles shaped starting at developmental patterning programs and further refined by local neuronal circuits throughout life. While astrocyte specialization has been more characterized in regions such as the cortex, spinal cord, hippocampus, and striatum, thalamic astrocytes have not been as thoroughly defined. Given the thalamus’ central role in sensorimotor integration and its known vulnerability following TBI^48^, alterations in astrocyte proteins may reflect region-specific functional adaptations or impairments.

Recently, studies have further demonstrated substantial transcriptional heterogeneity of the astrocyte population across brain regions as well, with specific focus on hippocampus and cortex^49,50^. While functional heterogeneity still remains to be fully explored, intraregional astrocytic heterogeneity is further thought to be established based on interactions with local neuronal subtypes, such as differing electrophysiological properties^51^.

Our previous studies found astrocyte morphological changes within the thalamic domain, but no significant differences within the cortex following a mild TBI and subsequent bup administration^38,39^. While it has been demonstrated that astrocytes express all three opioid receptors) and are responsive to opioid treatment^52,53^, little is known about regional differences on opioid-driven astrocyte changes. This study, in turn, examined how a single dose of a sustained-release bup formulation, bup-SR-LAB, a commonly used analgesic in pre-clinical TBI animal models, affects the regional expression of key astrocytic proteins following a single, diffuse, mild TBI. Specifically, we assessed markers of cytoskeletal remodeling and reactivity (GFAP) and water and ion homeostasis (AQP4, TRPM4, and SWELL1/LRRC8A) at 1 day and 4 weeks following injury. Our results demonstrate several region- and time-dependent changes after a central fluid percussion injury (cFPI) with selective modulation by bup, suggesting that even a single post-injury opioid exposure can influence astrocytic reactivity, edema regulation, and fluid clearance beyond the more acute phase of injury response.

GFAP protein levels were elevated in saline-treated animals following mTBI relative to sham controls in the current study (Fig. 1). Our findings are consistent with previous studies demonstrating increased GFAP expression in brain tissue following a TBI. For example, certain studies have found increased GFAP immunoreactivity anywhere from 7 hours to 35 years in human post-mortem brain cases^54^. Astrocyte activation has also been detected by GFAP immunohistochemistry up to one year following cortical impact injury in mice^55^, and increased astrocytic GFAP immunoreactivity has been observed in post-mortem human TBI tissue using an immunohistochemical and multidimensional MRI approach^56^.

In the current study, we have also observed region-specific differences in GFAP protein expression levels. At 1-day post-TBI, GFAP expression in the hippocampus and thalamus was greater than that in the cortex regardless of bup treatment (Fig. 1). By 4 weeks post-TBI, hippocampal GFAP expression remained higher than cortical expression in saline-treated injured animals, with no significant difference seen between hippocampus and thalamus. These findings are consistent with previous literature demonstrating region-specific differences in astrocyte number and GFAP expression, with phylogenetically older brain regions, such as the limbic cortex, exhibiting more intense GFAP-positive astrocytic staining^57–60^. Further, a recent single-cell RNA sequencing and spatial localization study reported substantially higher GFAP mRNA expression in the hippocampus compared to the cortex of adult mice^50^. Although relatively few studies have examined thalamic astrocytic changes, evidence suggests that thalamic astrocytes develop earlier than cortical astrocyte populations^61^, which may contribute to the elevated baseline GFAP levels observed in thalamus relative to cortex in our control animals. Notably, astrocytic responses following TBI appear to evolve over a prolonged time course, additionally seen in our current study. Previous work investigating GFAP staining in a rat cortical impact model reported a delayed onset and prolonged elevation in GFAP immunoreactivity compared to the more rapid and transient profile observed for microglial markers such as CD11b and Iba1^62^.

In contrast to saline-treated animals, the injury-associated increase in GFAP was not observed in bup-treated animals (Fig. 1). Specifically, we detected a marked decrease in GFAP expression in the hippocampus of animals receiving both TBI and buprenorphine relative to saline-treated TBI animals (Fig. 1). These findings suggest that TBI-induced increases in GFAP expression are impaired by acute opioid exposure, particularly within the hippocampus, pointing to a regional susceptibility. These findings align with previous work from our group demonstrating that a single dose of bup alters astrocyte morphology following diffuse TBI^38,39^ and support the concept that astrocytic responses evolve dynamically in both region- and injury-dependent ways. This finding is also consistent with previous studies reporting reduced GFAP expression following opioid treatment^63^. However, other studies have reported that sustained opioid treatment over multiple days can increase GFAP expression^64^, potentially through signals sent by neurons^65^, highlighting the complex interplay among neural cell types following opioid exposure.

SWELL1/LRRC8A is a pore-forming subunit of the volume-regulated anion channel (VRAC), which mediates chloride and organic osmolyte, including glutamate, efflux out of the cell during regulatory volume decreases in a multitude of cells including astrocytes, endothelia, and microglia^66^. It is a key component to controlling cellular swelling by removing osmotic molecules from the cell, thus rescuing cells from oncosis^28^. More specifically, SWELL1 is a four-transmembrane protein localized to the plasma membrane that is shown to be essential for hypotonicity-induced iodide influx^24^. Thus, in the context of post-TBI fluid regulation, this VRAC channel complex is critical to study, particularly when in the context of the brain’s response to drugs. Not directly in opioid studies, but recent findings have furthered the importance of the SWELL1 subunit of the VRAC as critical in astrocyte function in regulating dopaminergic circuitry and cocaine reward^67,68^. In this study, we observed significantly lower SWELL1 expression in the thalamus compared to either the hippocampus or cortex in sham control animals treated with saline (Fig. 2), indicating intrinsic regional differences in SWELL1 expression. These regional differences were lost with bup treatment, with all three tested regions (cortex, thalamus, hippocampus), suggesting relatively equivalent SWELL1 expression levels after bup administration in sham injury (Fig. 2). Interestingly, analysis of the TBI groups indicated lower cortical expression but increased thalamic expression of SWELL1 at a 4-week timepoint following injury and bup administration (Fig. 2). The hippocampus showed no significant changes across TBI treatment groups. These observations suggest enhanced regional susceptibility to opioid modulation of SWELL1 following an injury, since these changes were not observed in our sham control rats.

While comparative regional evaluations of SWELL1/LRRC8A expression are limited, studies have linked SWELL1 to post-injury-associated neurotoxicity. Increases in SWELL1 expression have been shown in lesions following ischemia^69^. After injury, increases in VRAC/SWELL1 signaling have been shown to influence astrocytic swelling and modulate the release of excitatory or inflammatory mediators^24,70^, linking it directly to acute and chronic brain volume disturbances and subsequent neurotoxicity. Knock out of SWELL1 specifically in astrocytes has been shown to reduce pathology and behavioral morbidity following ischemic stroke linked to reduced neuronal excitability due to lower levels of ambient glutamate released from astrocytes through the SWELL1 channel^71,72^. Activation of SWELL1 in microglia, however, has been shown to reduce neuroinflammation and overall brain damage, indicating the impact of SWELL1 activation is highly cell dependent^64,73^. Finally, while a previous study found that cocaine administration activated SWELL1^68^, this appears to be the first study to specifically investigate the impact of opioid administration on SWELL1 expression in various brain regions.

TRPM4 is a nonselective calcium-activated cation channel implicated in astrocytic swelling and barrier dysfunction. Specifically, TRPM4 channels regulate water movement into the cell by allowing influx of osmotic cations which induces swelling. Following injury, TRPM4 contributes to osmotic dysregulation, cell swelling, and blood-brain barrier disruption. Its activation amplifies astrocytic and endothelial vulnerability under conditions of ionic and metabolic stress^74–76^. While previous studies indicate that injury induces elevated TRPM4 expression^26,75,76^, with specific focus on either the hippocampus or cortex, there is limited information regarding regional heterogeneity in TRPM4 expression, especially in TBI disease contexts. A recent murine study found that TRPM4 plays a pivotal role in regulating the astrocytic Ca^2+^ signaling, medicating aberrant expression and subcellular localization of the more well-studied astrocytic AQP4 along perivascular pathways^77^. However, no studies currently explore the impact of opioids on TRPM4 expression in physiologically normal or TBI-injured brains.

In sham control animals, no regional differences between cortex, thalamus, or hippocampus were observed (Fig. 3), suggesting TRPM4 levels to be more consistent across brain regions in the healthy brain. Within the saline-treated TBI cohorts, however, TRPM4 expression was higher in the cortex and hippocampus relative to the thalamus (Fig. 3). This finding is congruent with previous findings of TRPM4 elevation in both the hippocampus and cortex following injury^26,76,78^. Interestingly, this injury effect was absent in bup-treated TBI animals primarily due to a significant reduction in TRPM4 levels in the cortex at 4 weeks following TBI and bup administration (Fig. 3). Our findings preliminarily indicate that bup also modifies regional TRPM4 regulation after injury, with a particular susceptibility of the cortex regarding expressed levels of TRPM4.

The principal astrocytic water channel, AQP4, is a bidirectional channel that allows for passive osmotic gradient-directed diffusion of water molecules across cellular membranes^27^. It is expressed on astrocytic endfeet associated with vasculature throughout the brain and has been shown to associate with both SWELL1 and TRPM4, which drive the osmotic gradients^28,75^. As referenced previously, recent studies have found dysregulation of endfeet-localization of AQP4 in TRPM4-knockout mice in the context of status epilepticus, underscoring the interplay with these water-regulating molecules and astrocytes in brain disease states^77^. And while much more abundant in astrocytes and often used as a marker of astrocytes, AQP4 has also been found in other cells, such as ependymal cell membranes and in osmosensory areas of the hypothalamus^79^. In previous rat TBI studies, AQP4 has been found to be downregulated during periods of vasogenic edema but conversely upregulated during cellular edema, suggesting that the upregulation of AQP4 may have induced cellular edema^80^.

In the current study, we found significant regional differences in AQP4 expression with the thalamus expressing the highest AQP4 levels and the cortex conversely expressing the lowest when looking at sham saline controls (Fig. 4B). This finding is consistent with previous work that found significantly higher levels of AQP4 immunolabeling in the thalamus and hippocampus compared to the cortex^81^. Acutely following injury, regional differences shift, with lower thalamic AQP4 expression relative to either the cortex or hippocampus (Fig. 4A). However, 4 weeks post-TBI, AQP4 levels are relatively consistent across brain regions with a significant increase in cortical AQP4 following TBI that was not seen in either the hippocampus or thalamus (Fig. 4B). Previous studies are mixed regarding the impact of TBI on AQP4 expression, with some studies finding overall increases in AQP4 expression days following TBI that returned to normal levels within a week post-TBI^82^ while others found reductions in AQP4 following TBI, particularly in perivascular areas^83,84^. These differences could be due to variability in the regions assessed and injury modality, however, they further support the concept that AQP4 levels are appreciably impacted following TBI.

While bup treatment did not significantly impact AQP4 expression in sham controls, there was a trend toward reduced expression in the thalamus of sham-injured rats treated with bup at 4 weeks (Fig. 4B). Interestingly, in the TBI animals, both hippocampal and thalamic AQP4 was significantly reduced following the bup dose, indicating a potential interaction between diffuse brain injury and opioid treatment (Fig. 4B). These findings suggest that bup alters the normal progression of AQP4 regulation after injury, particularly decreasing hippocampal and thalamic AQP4 expression at later stages of recovery post-TBI, an effect not observed in the cortical expression levels. AQP4 and μ-opioid receptors have been shown to coprecipitate and morphine treatment of cultured astrocytes decreased AQP4 expression supporting the potential of direct impacts of opioids on AQP4^85^. Self-administration and other addiction-related effects have also been observed to decrease in transgenic mice lacking AQP4^86,87^, indicating a modulatory role of AQP4 in mediating opioid outcomes.

Recent studies demonstrated that AQP4 is necessary for proper interstitial fluid dynamic clearance of solutes from the brain via the glymphatic system^81,88,89^. Alterations in astrocytic water channel expression have also been associated with changes in parenchymal fluid dynamics following diffuse brain injury^83,90^. Therefore, to explore a functional correlate of these molecular changes, we examined thalamic dextran retention at 4 weeks following TBI in saline or bup-treated animals. We did not observe effects of bup in sham animals, but we did observe a trend toward higher thalamic dextran burden with bup treatment compared to saline following TBI, indicating potential bup-associated alterations in solute clearance (Fig. 5). A recent study using diffuse tensor imaging found significant glymphatic system alterations in people suffering from heroin addiction^91^, supporting the potential that opioids impair glymphatic function.

Previous studies have shown that injury induces AQP4 water channel mislocalization or dysregulation, contributing to cytotoxic edema and impaired glymphatic clearance and astrocyte-dependent waste removal^92,93^. In this study, we further examined TBI thalamic tissue for potential alterations in the spatial relationship between AQP4 and the astrocytic cytoskeletal marker GFAP using an intensity-based colocalization approach. Across animals, measures of AQP4 signal colocalized with GFAP were highly variable and did not segregate cleanly by treatment condition, suggesting that diffuse TBI produces heterogeneous astrocytic remodeling rather than a uniform shift in AQP4-GFAP association. While some bup-treated animals exhibited relatively higher fractions of AQP4 signal proximal to GFAP, this pattern was inconsistent and overlapped substantially with saline-treated animals, limiting inference of a treatment-dependent effect on astrocytic localization. These findings indicate that changes in bulk AQP4 expression and thalamic dextran retention observed at 4 weeks are not necessarily accompanied by a consistent redistribution of AQP4 relative to GFAP-positive processes. Instead, bup-associated effects on water handling after diffuse TBI may reflect altered channel abundance, regulation, or activity rather than dysregulated localization. The substantial inter-animal variability underscores the dynamic and heterogeneous nature of astrocytic responses after diffuse brain injury. It is also possible that this variability is due to the fact that we assessed the entire hemi-thalamus rather than focusing on specific nuclei, as the thalamus contains multiple distinct nuclei and regions with traversing white-matter tracts.

This study has several limitations. We examined only male rats and used a single post-injury bup dose. Sex differences in TBI pathophysiology and glial activation are increasingly recognized^94–97^, therefore future studies including females would be essential for fully elucidating these effects in the greater population. Further, western blotting measures bulk tissue and cannot resolve astrocyte subpopulations, subcellular compartments such as perivascular versus parenchymal domains, or different thalamic nuclei being differentially affected. While we did investigate AQP4 as it associates with GFAP-positive astrocyte processes, perivascular changes may exist in the various channels investigated that were not resolved in the current study. As we assessed only two time points, we may have missed more acutely transient or later-phase changes in the various astrocyte-associated channels following TBI and bup treatment. Adding earlier and more chronic sampling would help clarify whether bup delays resolution or induces lasting alterations. Additionally, sham cohorts were not included in the 1-day aspect of this study, which limits interpretation of early injury × treatment interactions. Finally, other analgesics, including full opioid agonists and non-opioid alternatives, also affect glial and inflammatory pathways and would warrant investigation^98^.

Overall, these findings demonstrate that even a single post-injury buprenorphine exposure can shape regional astrocytic protein expression weeks after diffuse TBI and may relate to altered thalamic fluid clearance. These molecular findings coincide with preliminary observations of greater thalamic dextran retention in bup-treated TBI animals, suggesting the possibility that opioid exposure changes astrocytic water and ion homeostasis in a way that may interfere with astrocytic water and ion homeostasis and, in turn, glymphatic clearance. As the field moves toward multidimensional TBI classification that incorporates modifiers such as medication exposure, recognizing and accounting for drug-induced alterations in glial physiology will be crucial for experimental design, data interpretation, and clinical translation to the human population.

## Methods

### Animals

For this study, a total of 39 adult (12–16-week-old) male Sprague-Dawley rats stratified by timepoint, injury, and treatment condition were utilized. No rats met pre-determined humane endpoints prior to scheduled experimental timepoints. Assessments using these animal cohorts have been published previously^38–40^. All studies were performed following ARRIVE guidelines^41^ and were approved by the Institutional Animal Care and Use Committee at Virginia Commonwealth University (VCU) (approval: AM10251). Studies followed all VCU institutional ethical guidelines regarding care and use of laboratory animals as well as guidelines in the *Guide for the Care and Use of Laboratory Animals: 8th Edition*^42^. Animals were housed in individual cages on a 12-hour light-dark cycle, had free access to food and water, and received full veterinary oversight throughout the study. Rats were randomized into groups using a random number generator prior to surgeries. Groups for the 1-day cohort were: (1) TBI with saline treatment (*n* = 6) and (2) TBI with bup-SR-LAB treatment (*n* = 5). Groups for the 4-week cohort were: (1) sham with saline treatment (*n* = 6), (2) sham with bup-SR-LAB treatment (*n* = 7), (2) TBI with saline treatment (*n* = 7), and (4) TBI with bup-SR-LAB treatment (*n* = 6). Tissue was also collected from two naïve animals for use as protein quantification normalization controls.

### Surgical Preparation, Injury Induction, and Drug Administration

All animals underwent surgical preparation and either sham-injury or TBI induction in a manner previously reported by our lab. Anesthesia was induced with 4% isoflurane in 30% oxygen/70% room air. Animals were ventilated with 1.5–2.5% isoflurane in 30% oxygen/70% room air throughout the surgery. Body temperature was maintained at 37°C using a rectal thermometer connected to a feedback-controlled heating pad (Harvard Apparatus, Holliston, Massachusetts, United States). Animals were placed in a stereotaxic frame (David Kopf Instruments, Tujunga, California, United States), and a 4.8 mm diameter circular craniectomy was made along the sagittal suture midway between bregma and lambda while leaving the dura intact. Subsequently, a 2 mm diameter burr hole was drilled over the left lateral ventricle positioned at 1.3 mm lateral, 0.8 mm posterior, and 2.5 to 3 mm ventral to bregma. A 25G canula connected to a pressure transducer and an 11 Elite microinfusion pump (Harvard Apparatus) was positioned in the lateral ventricle to measure pre-injury intracranial pressure (ICP) as reported in^38,39^. Bone wax was used to seal the burr hole used for ICP measurements before the induction of either sham-injury or TBI.

For induction of diffuse TBI via central fluid percussion injury (cFPI), a Luer-Lok^™^ syringe (BD, Franklin Lakes, New Jersey, United States) hub was connected to the craniectomy site with cyanoacrylates and methyl methacrylate (Hygenic, Akron, Ohio, United States). Once the methyl methacrylate hardened, animals were removed from the frame and connected to the fluid percussion device to complete a closed fluid-filled system from the intact dura of the animal to the end of a Plexiglas cylinder. To induce a mild-to-moderate cFPI, a pendulum was released onto the end of the fluid-filled cylinder of the percussion device, producing a pressure pulse that was transduced through the intact dura to the CSF and spread diffusely throughout both hemispheres of the brain. The pressure pulse reached the animal at 2.05 ± 0.10 atm and lasted roughly 22.5 ms measured via a transducer affixed to the injury device and displayed on an oscilloscope (Tektronix, Beaverton, Oregon, United States). For sham-injured animals, the same procedures were performed minus the pendulum release. Immediately following cFPI or sham injury, animals were reconnected to the ventilator, the hub, dental acrylic, and bone wax were removed, and gel foam was placed on the craniectomy site. The animals were replaced in the stereotaxic frame, and the ICP probe was reinserted into the lateral ventricle to establish post-injury ICP monitoring.

Fifteen min following either TBI or sham injury, either a veterinarian-recommended dose of extended-release bup (1 mg/kg of a 1 mg/mL bup-SR-LAB solution, ZooPharm, Laramie, Wyoming, United States) or equal volume of saline was administered subcutaneously to the animals. Following recovery, rats were returned to clean, individual home cages. Comprehensive postoperative monitoring of animals was done daily for the first three days post-injury followed by weekly checks thereafter for up to 4 weeks.

### Tracer Infusion

At 4 weeks post-injury, rats were anesthetized with 4% isoflurane and 30% oxygen/70% room air and maintained on 2% isoflurane and 30% oxygen/70% room air via nose cone for the duration of the tracer infusion. Rats were secured in the stereotaxic frame, the scalp was incised, and an ICP needle was filled with 0.7 mg/17 μL 10 kDa tagged dextran and inserted into the left lateral ventricle. ICP was monitored for 15 min post-needle placement, with the dextran then being infused at a rate of 1.3 μL/min while maintaining ICP at normal ranges. Tracer was given two hours for both diffusion throughout the brain parenchyma and clearance prior to sacrifice^38,39^.

### Animal Sacrifice and Tissue Processing

At either 1 day or 4 weeks post-injury, and 2 hours following tracer infusion, anesthetized rats were overdosed with pentobarbital sodium and phenytoin sodium (Euthasol^®^ euthanasia-III solution, Henry Shein, Dublin, Ohio, United States) followed immediately by transcardial perfusion first with cold 0.9% saline then with 4% paraformaldehyde/0.2% glutaraldehyde in Millonig’s buffer (136 mM sodium phosphate monobasic/109 mM sodium hydroxide). Fresh right-hemisphere tissue and perfusion-fixed left-hemisphere tissue were collected from each rat, as previously described^38,39^.

Briefly, following transcardial perfusion with cold 0.9% saline, a tissue core of right lateral neocortex, hippocampus, and thalamus were taken for protein expression quantification. Once fresh brain samples were collected from the right hemisphere, animals were transcardially perfused with 4% paraformaldehyde/0.2% glutaraldehyde in Millonig’s buffer (136 mM sodium phosphate monobasic/109 mM sodium hydroxide) for preservation for immunohistochemical analyses of the left hemisphere. Both hemispheres were usable for analysis because of the bilateral nature of the cFPI model of diffuse TBI. Fixed tissue from the left hemisphere was then removed and post-fixed for at least 72 hours. Fixed brain tissue was coronally sectioned at 40 μm in 0.1 mM phosphate buffer using a vibratome (Leica, Bannockburn, Illinois, United States) from bregma to roughly 4 mm posterior to bregma. Sections were collected serially in 12-well plates and stored in Millonig’s buffer at 4°C prior to use.

### Quantification of Protein Expression

To assess the expression of target proteins, western blotting was performed. Each sample was run in triplicate on separate gels and averaged to minimize potential run-to-run variability significantly biasing results. Rat tissue from the right lateral neocortex, hippocampus, and thalamus was dissected and homogenized in either Stabilyser Reagent (cat#: PNS1010, Sigma Aldrich, Saint Louis, Missouri, United States) or in NP40 buffer (150 mM NaCl, 50 mM Tris pH 8.0, 1% TritonX) and protease inhibitor cocktail (AEBSF 10.4 mM, Aprotinin 8 μM, Bestatin 400 μM, E-64 140 μM, Leupeptin 8 μM, Pepstatin A 150 μM, cat#: P8340, Sigma Aldrich, Saint Louis, Missouri, United States). Protein concentration was determined via a Pierce^™^ BCA Protein Assay Kit (cat#: 23225, Thermo Fisher Scientific, Waltham, Massachusetts, United States) according to manufacturer instructions and quantified on a PHERAstar spectrophotometer (BMG Labtech, Cary, North Carolina, United States).

Samples for each region of interest were prepared identically for each run. 5 μg of protein from each sample was boiled for 10 min at 95°C in 1X Laemmli Sample Buffer (cat#: 1610737, BioRad Laboratories, Hercules, California, United States), and 1 M dithiothreitol (DTT) to reduce disulfide bonds. Samples were run at 100 V for 5 min (to ensure consistent front) then at 200 V for 30 min on Mini-PROTEAN^®^ TGX Stain-Free Precast Gels (4–20% polyacrylamide, cat#: 4568096, BioRad Laboratories) in 10X Tris/Glycine/SDS running buffer (cat#: 1610732, BioRad Laboratories) using a Mini-PROTEAN^®^ Tetra Vertical Electrophoresis Cell (cat#: 1658004, BioRad Laboratories) housing. Protein was transferred onto 0.45 μm low-fluorescent PVDF membranes via a Trans-Blot^®^ Turbo^™^ Transfer System (cat#: 1704150, BioRad Laboratories) on the pre-programmed “Mixed Molecular Weight” setting (2.5 Amps and 25 V for 7 min).

Total protein in each lane was visualized using either the stain-free tryptophan marker imaged using a UV light on a ChemiDoc^™^ Touch (BioRad Laboratories) or the Total Protein Q fluorescent protein stain (Azure Biosystems Cat. # AC2225) imaged using an Azure Q600 (Azure Biosystems, Dublin, California, United States) imager. Western blotting was performed on an iBind^™^ Flex Western Device (Invitrogen^™^, Thermo Fisher Scientific, Waltham, Massachusetts) using primary and species-appropriate secondary antibodies as listed in Table 9 GFAP, SWELL1/LRRC8A, and TRPM4 were visualized using HRPconjugated secondary antibodies followed by chemiluminescent imaging (ChemiDoc^™^ Touch) while AQP4 was visualized using an IR800 secondary antibody followed by fluorescent imaging (Azure Q600).

Bands of interest for each protein were quantified via densitometric analysis (integrated density) in Fiji ImageJ (National Institutes of Health, Bethesda, Maryland, United States) and normalized to membrane background manually, as openly reported in the Open Science Framework (OSF; DOI 10.17605/OSF.IO/8RGUQ.). For GFAP, the single band at ~ 50 kDa was quantified. For SWELL1/LRRC8A the doublet at ~ 85–105 kDa was quantified together. For TRPM4, because consistent banding was observed throughout, the prominent bands at ~ 30, 55, 85, and 200 kDa were quantified and summed for analysis, noting the difference between in-house and manufacturer observed molecular weight. For AQP4 the single band at ~ 28 kDa was quantified. All sample values were normalized to total protein in each lane. The total protein normalized values were then normalized to the naïve animal sample, which was run alongside the study samples on each gel and this value was used for statistical analysis.

### Immunohistochemistry

Thalamic tissue from the 4-week post-injury cases was assessed for changes in dextran clearance and the distribution of the water channel, AQP4. For immunohistological assessments, two sections were imaged per each case. As the right hemisphere of the brain was collected for fresh tissue molecular assessments, only the left hemi-thalamus was imaged for all immunohistological assessments reported in this study.

To assess the distribution of the AQP4 water channels, we performed immunostaining against AQP4 using the same primary antibody used for western blot assessments (Table 9), followed by goat anti-rabbit secondary antibodies conjugated to either Alexa-488 or Alexa-568 depending upon the fluorescently tagged dextran infused (Table 9). Astrocyte cytoskeletal structure was visualized using an Alexa-647 tagged mouse anti-GFAP antibody (Table 9). Tiled z-stacks of the entire left hemi-thalamus for each 4-week TBI case were captured using a ZEISS (Oberkochen, Germany) Elyra 7 structured illumination microscope (SIM), equipped with a 25x oil objective (NA 0.8) and two sCMOS cameras (Orca Flash, Hamamatsu). Fluorescence of AQP4 was excited with a 561 nm laser (7.5% nominal power) and detected in the 570–625 nm range. GFAP fluorescence was imaged with 634 nm excitation (910% power) and a 640–700 nm emission range, respectively. Raw images (voxel size 0.15 μm lateral and 0.42 μm axial) were collected at 40 ms acquisition time in both channels. The 3D (z-stack) SIM datasets were reconstructed through a combination of raw images in frequency space with Wiener filtering (signal-to-noise ratio of 6). The reconstructed images were further refined using constrained deconvolution (Tikhonov-Miller) with 7 or 11 iterations and a regularization factor of 0.075 or 0.125, depending on the channel. Individual voxels from Nyquist-sampled (every 10th x-,y-frame and every 5th z-plane) reconstructed images were extracted using MATLAB^®^ (MathWorks, Natick, Massachusetts), excluding z-frames with manually identified artifacts that would bias the analysis. A low-pass filter for each 13-pixel neighborhood was used to reduce analysis of low-intensity background pixels. Colocalization was assessed for AQP4-positive and GFAP-positive pixels using FlowJo^™^ (*v10.1*, Becton, Dickinson & Company, Ashland, Oregon). Grey value intensity thresholds were set at 892 for AQP4 and 1245 for GFAP, and they were based on the average pixel grey value visually identified as true signal from each 16-bit tiled 3D stack. Colocalization coefficients were averaged between the two sections imaged for each case.

To investigate the clearance of intracerebroventricularly-infused 10 kDa dextran, images of the left thalamus were captured using a 2x objective on a BZ-800 Keyence microscope system (Keyence Corporation, Osaka, Japan). Imaging settings were held consistent for all slides imaged. The thalamus was isolated and the percent of the region of interest containing the dextran was quantified using a consistent intensity threshold.

### Statistics

Statistical analyses for western blots and histological data were performed in R (*v4.5.1*, R Foundation for Statistical Computing, Vienna, Austria). Factorial analysis of variance (ANOVA) was used for between group comparisons. Two-way ANOVAs (region × treatment) were done for 1-day comparisons, and three-way ANOVAs (injury × region × treatment) were done for 4-week comparisons. When significant main effects or interactions were detected, post-hoc pairwise comparisons within relevant cohorts were performed using Welch’s t-tests with Benjamini-Hochberg (BH) correction for multiple comparisons where appropriate. Longitudinal effects across 1 day and 4 weeks were assessed using repeated-measures ANOVA including timepoint as a fixed factor. Significance was set as *p* < 0.05 after correction where applicable.

## Supplementary Material

Supplementary Files

This is a list of supplementary files associated with this preprint. Click to download.

- supplementaryfile.docx
- FullBupAstrocyteData.xlsx

## Figures and Tables

**Table 1:** 1 Day GFAP Two-Way ANOVA Results (region, treatment).

Factor	df_1_	df_2_	*F*	*p*	Significance
region	2	25	11.27	<0.001	***
treatment	1	25	0.04	0.843	**ns**
region × treatment	2	25	0.01	0.989	**ns**

**Table 2:** 4 Weeks GFAP Three-Way ANOVA Results (injury, region, treatment).

Factor	df_1_	df_2_	*F*	*p*	Significance
injury	1	64	6.17	0.016	*
region	2	64	12.76	<0.001	***
treatment	1	64	1.34	0.251	**ns**
injury × region	2	64	2.73	0.073	.
injury × treatment	1	64	7.25	0.009	**
region × treatment	2	64	0.11	0.898	**ns**
injury × region × treatment	2	64	1.28	0.284	**ns**

**Table 3:** 1 Day SWELL1 Two-Way ANOVA Results (region, treatment).

Factor	df_1_	df_2_	*F*	*p*	Significance
region	2	24	0.49	0.618	**ns**
treatment	1	24	0.57	0.458	**ns**
region × treatment	2	24	1.86	0.178	**ns**

**Table 4:** 4 Week SWELL1 Three-Way ANOVA Results (injury, region, treatment).

Factor	df_1_	df_2_	*F*	*p*	Significance
injury	1	64	0.734	0.395	**ns**
region	2	64	10.683	<0.001	***
treatment	1	64	0.678	0.413	**ns**
injury × region	2	64	1.406	0.253	**ns**
injury × treatment	1	64	3.286	0.075	.
region × treatment	2	64	3.300	0.043	*
injury × region × treatment	2	64	2.518	0.087	.

**Table 5:** 1 Day TRPM4 Two-Way ANOVA Results (region, treatment).

Factor	df_1_	df_2_	*F*	*p*	Significance
region	2	24	13.21	<0.001	***
treatment	1	24	0.40	0.534	**ns**
region × treatment	2	24	0.24	0.793	**ns**

**Table 6:** 4 Week TRPM4 Three-Way ANOVA Results (injury, region, treatment).

Factor	df_1_	df_2_	*F*	*p*	Significance
injury	1	64	3.765	0.057	.
region	2	64	4.996	0.010	**
treatment	1	64	0.091	0.764	**ns**
injury × region	2	64	0.253	0.777	**ns**
injury × treatment	1	64	0.589	0.446	**ns**
region × treatment	2	64	1.991	0.145	**ns**
injury × region × treatment	2	64	4.651	0.013	*

**Table 7:** 1 Day AQP4 Two-Way ANOVA Results (region, treatment).

Factor	df_1_	df_2_	*F*	*p*	Significance
region	2	24	34.90	<0.001	***
treatment	1	24	0.25	0.619	**ns**
region × treatment	2	24	0.60	0.555	**ns**

**Table 8:** 4 Week AQP4 Three-Way ANOVA Results (injury, region, treatment).

Factor	df_1_	df_2_	*F*	*p*	Significance
injury	1	63	0.318	0.575	**ns**
region	2	63	14.303	<0.001	***
treatment	1	63	8.714	0.004	**
injury × region	2	63	6.555	0.003	**
injury × treatment	1	63	0.891	0.349	**ns**
region × treatment	2	63	3.059	0.054	.
injury × region × treatment	2	63	2.107	0.130	**ns**

**Table 9:** Primary and secondary antibodies and dilutions used for western blotting.

Antibody	Host SPecies	Dilution	ComPany	Catalog #	Molecular Weight (kDa)
AQP4	Rabbit	1:1,000	Cell Signaling Technology	59678	28
GFAP	Mouse	1:10,000	MilliPore Sigma	MAB3402	50
LRRC8A/SWELL1	Rabbit	1:1,000	Cell Signaling Technology	24979	85-105
TRPM4	Rabbit	1:1,000	Boster Biological Technology	PB9902	Calculated: 134 Observed: 167
Anti-Rabbit IgG (H + L)	Goat	1:5,000	Jackson ImmunoResearch Laboratories	111-035-003	--
Anti-Mouse IgG (H + L)	Goat	1:5,000	Jackson ImmunoResearch Laboratories	115-035-003	--
Anti-Rabbit IgG IR800	Goat	1:2,000	Azure Biosystems	AC2134	--

## Data Availability

Data used for all analyses done in this work are appended as a supplemental document. Data is also publicly available on the Open Science Framework DOI:10.17605/OSF.IO/7WV4B (https://osf.io/7wv4b/overview).
